# Bergey’s Principles of Hygiene

**Published:** 1902-04

**Authors:** 


Bergey's Principles of Hygiene.—The principles of hygiene A practical manual for students, physicians, and health officers. By D. H. Bergey, A. M., M. D., First Assistant, Laboratory of Hygiene, University of Pennsylvania. Octavo volume of 495 pages, illustrated. Philadelphia and London: W. B. Saunders & Company. 1901. Cloth, $3.00 net.

Among the subjects treated of in this work are air, ventilation, heating, water supply, sewage and garbage disposal, diet, exercise, clothing, personal hygiene, school hygiene, soil, habitations, disinfection, quarantine, and vital statistics. Although Dr. Bergey does not pretend to treat of these subjects in an exhaustive manner, he gives all of the facts that students, physicians and health officers will find time to study or will be likely to need. The book is a worthy companion volume to Dr. A. C. Abbott's "Hygiene of the Transmissable Diseases." R.

